# SUBSIDIZED SENIOR HOUSING IN THE U.S.: A SCOPING REVIEW

**DOI:** 10.1093/geroni/igad104.3607

**Published:** 2023-12-21

**Authors:** Sojung Park, Soobin Park, Byeongju Ryu, Jihye Baek, Takashi Amano, BoRin Kim

**Affiliations:** Washington University in Saint Louis, St. Louis, Missouri, United States; Washington University in Saint Louis, St. Louis, Missouri, United States; Boston College, Boston, Massachusetts, United States; Washington University in Saint Louis, St. Louis, Missouri, United States; Rutgers University, Newark, New Jersey, United States; University of New Hampshire, Durham, New Hampshire, United States

## Abstract

Although Subsidized Senior Housing (SSH) has been considered an important model to promote healthy aging among vulnerable subgroups of older adults, progress in developing, expanding, and improving SSH has been limited compared to other housing models. A comprehensive review of the recent knowledge base is a critical step toward developing and expanding policies and programs for senior housing for low-income older adults. This scoping review used a five-stage scoping review methodology to answer two research questions: 1) What are the trends in SSH empirical studies? 2) What are the main topics in the existing literature on SSH in the US? This review explores the social, physical, and service environments related to SSH in older adults in the US. Personal and environmental factors were key in examining study outcomes or main focus. We reviewed a total of 61 articles and found several notable trends, including a focus on racial and ethnic minorities, cognitive impairment among SSH residents, and Collaborative partnerships between housing operators and healthcare providers. More than half of the studies (54%) were quantitative, while the rest were qualitative (38%) or mixed-methods (8%). The lack of longitudinal or causal design research reflects the limited empirical knowledge of SSH. Four themes emerged: health and well-being (52.5%), healthcare use and health behaviors (31.1%), social relations (8.2%), and housing relocation (8.2%). We discussed implications for future research and program development efforts in SSH. Future research should explore the fit between individual characteristics and the multiple-factor environmental context.

